# Role of cytochrome P450s in insecticide resistance: impact on the control of mosquito-borne diseases and use of insecticides on Earth

**DOI:** 10.1098/rstb.2012.0429

**Published:** 2013-02-19

**Authors:** Jean-Philippe David, Hanafy Mahmoud Ismail, Alexia Chandor-Proust, Mark John Ingraham Paine

**Affiliations:** 1 Laboratoire d'Ecologie Alpine, UMR 5553, CNRS-Université de Grenoble, BP 53, 38041 Grenoble cedex 09, France; 2 Liverpool School of Tropical Medicine, Liverpool L3 5QA, UK

**Keywords:** mosquito, malaria, dengue, pyrethroids, oxidases, detoxification

## Abstract

The fight against diseases spread by mosquitoes and other insects has enormous environmental, economic and social consequences. Chemical insecticides remain the first line of defence but the control of diseases, especially malaria and dengue fever, is being increasingly undermined by insecticide resistance. Mosquitoes have a large repertoire of P450s (over 100 genes). By pinpointing the key enzymes associated with insecticide resistance we can begin to develop new tools to aid the implementation of control interventions and reduce their environmental impact on Earth. Recent technological advances are helping us to build a functional profile of the P450 determinants of insecticide metabolic resistance in mosquitoes. Alongside, the cross-responses of mosquito P450s to insecticides and pollutants are also being investigated. Such research will provide the means to produce diagnostic tools for early detection of P450s linked to resistance. It will also enable the design of new insecticides with optimized efficacy in different environments.

## 1. Introduction

Insect vectors are responsible for almost 20 per cent of all infectious diseases affecting people in developing countries [1]. This review focuses on mosquitoes as they are the commonest disease carriers for a host of parasites such as bacteria and viruses in man. Three species that are particularly important to man are *Anopheles gambiae*, which transmits malaria, *Aedes aegypti*, which transmits dengue and other viruses, and *Culex quinquifasciatus*, a vector for West Nile virus and other viral encephalitides. Their medical importance is reflected by the fact that they were among the first wave of genomes to be sequenced, *An. gambiae* in 2002 [2], *Ae. aegypti* in 2007 [3] and *C. quinquifasciatus* in 2010 [4]. This has revealed an extensive repertoire of P450s (more than 100) allowing the development of new tools for investigating the functional role of mosquito P450s and their relationships with insecticide metabolism.

The fight against diseases spread by mosquitoes has enormous environmental, economic and social consequences. Chemical insecticides remain the first line of defence but the control of disease is being undermined by resistance. P450s have been fundamental to the successful adaptation of insects to diverse habitats [5,6], thus the highly evolved P450 repertoire of mosquitoes provides excellent protection against insecticides and other xenobiotics, a subject that has been well reviewed [5,7,8]. However, there is limited knowledge about which P450s are responsible for insecticide resistance in mosquitoes. Furthermore, given their primary role in defence against plant and environmental toxins, it is unclear to what extent pollutants and other xenobiotics might influence the adaptation of the mosquitoes' P450 repertoire to its chemical environment and modulate insecticide resistance mechanisms. Filling-in these knowledge gaps will have important impact on the use of insecticides to improve efficiency and resistance management strategies as discussed in this review.

## 2. Diseases spread by mosquitoes

Mosquitoes transmit many diseases including malaria, dengue fever, Japanese encephalitis virus, West Nile virus, yellow fever virus and filariasis. Of these, malaria transmitted primarily by *An. gambiae*, dengue transmitted by *Ae. aegypti* and lymphatic filariasis transmitted by *C. quinquifasciatus* are the most devastating problems in terms of the global number of people affected. Approximately half of the world's population are at risk of malaria, with 225 million cases being recorded in 2009 [9]. Dengue fever, a flavivirus transmitted by *Aedes* mosquitoes (mainly *Ae. aegypti* and *Ae. albopictus*), is an increasingly serious public health problem in over 100 countries with some 2.5 billion people at risk [10]. *Culex quinquefasciatus* is a major biting nuisance in the urban tropics, and is a vector for West Nile virus in addition to lymphatic filariasis, which affects 120 million people worldwide, and can lead to genital damage and elephantiasis. Apart from the obvious health issues, the economic and environmental costs are high. Such diseases have detrimental effects on fertility, population growth, saving and investment, worker productivity and absenteeism, premature mortality and medical costs. Indeed, it has been estimated that every year malaria causes a gross domestic product (GDP) loss of $US 12 billion to Africa  [11]. Thus, countries with high malaria transmission have had historically lower economic growth than countries without malaria.

## 3. Insecticides

Given the obvious environmental impact of chemicals, why, in the twenty-first century, are we still using insecticides? In short, because for many vector-borne diseases they remain the cheapest, most effective method available. In the case of dengue, insecticides are the only realistic option for disease control as there are no clinically approved vaccines or antiviral therapies available [12]. In the case of malaria, drugs are available but there are problems of drug resistance and affordability [13–15]. Likewise, there are no clinically approved vaccines, although recent trials of a candidate malaria vaccine RTSS/AS01 are showing enough promise (approx. 50% protection) to suggest that vaccination will be a viable option in the foreseeable future [16]. Thus, targeting the vector remains the most effective option to significantly reduce disease transmission.

Environmental liabilities have, however, encouraged the development of natural alternatives with less side-effects. These include bacterial toxins from *Bacillus thuringiensis* var. *israelensis*, which are effective against mosquito larvae [17,18], and entomopathogenic fungi such as *Beauveria bassiana* and *Metarhizium anisopliae*, which are effective in killing adult mosquitoes [19–21]. There is also an escalating use of synthetic insect hormones to disrupt an insect's vital processes, such as growth or metamorphosis; and synthetic pheromones, powerful insect sex attractants, to sabotage pest reproduction and lure pests into traps. Finally, there is growing interest in the release of sterilized male insects to compete with fertile males for mates, diminishing the population of the next generation. The first open-field trial of transgenic *Ae. aegypti* in the Cayman Islands indicates that large-scale releases of mosquitoes to fight infectious diseases is feasible [22–24]. However, the high cost associated with this technique and the acceptance of genetically modified organism usage by stakeholders and populations may limit this approach.

Common chemical interventions include insecticide treated nets (ITNs), indoor residual spraying (IRS) and space spraying or fogging that target the adult stage (epidemiologically the most important life stage) [25]. Choice of application is largely dependent on mosquito behaviour. For indoor night biters such as *An. gambiae* and *C. quinquifasciatus,* bednets and IRS are effective. However, bednets and IRS have limited value against *Ae. aegypti* and *Ae. albopictus* as they are outdoor daytime biters; fogging and larviciding are generally used, which are less effective and logistically challenging.

**Figure 1.**
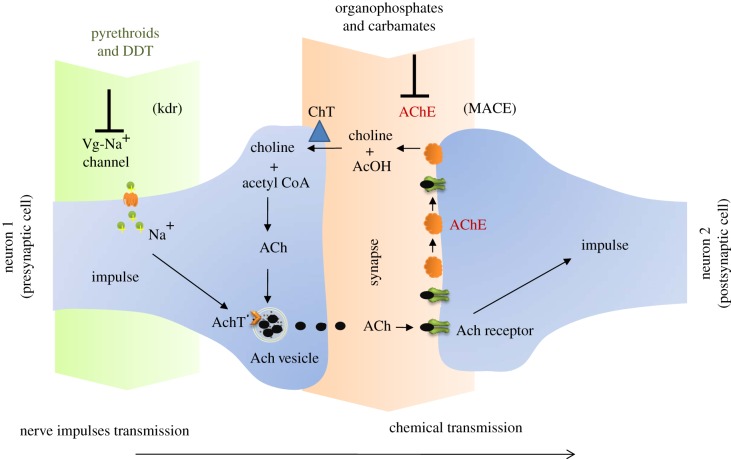
Biochemical target sites of synthetic insecticides. Pyrethroids and DDT exert their toxic effect by blocking the voltage-gated sodium channels, which generally produces fast knock-down properties (kdr). Organophosphate (OP) and carbamate insecticides inhibit acetylcholinesterase (AChE) which plays an important role in terminating nerve impulses. Reduced sensitivity of AChE as a result of a gene mutation (MACE) causes resistance to OP and carbamate insecticides (after [26]). ACh, acetylcholine; AchT, Ach transporter; AcOH, acetic acid; ChT, choline transporter; MACE, modified acetylcholinesterase; Vg-Na^+^ channel, voltage-gated sodium channel; kdr, knock-down resistance.

At present there are only four approved classes of insecticides (table 1) with only two modes of action, inhibition of the sodium channels (pyrethroids and DDT) or acetylcholine esterase (organophosphates and carbamates) (figure 1). This limited number plus the fact that no new insecticides have been approved for vector control by WHO in the last 30 years has greatly increased the threat of resistance and the sustainability of chemical insecticides for vector control. Pyrethroid insecticides are particularly vulnerable as they are the only insecticide class recommended for ITNs [27,28]. Between 2008 and 2010 more than 254 million ITNs have been delivered to malaria endemic countries in Africa [9,29,30], which has resulted in a significant reduction in the disease mortality and morbidity [31,32]. However, there has been a significant increase in reports of pyrethroid resistance in malaria vectors over the past decade [33,34]. Furthermore, such intensive use of long-lasting insecticide-treated nets (LLIN) and IRS against nocturnal indoor biting species of mosquito may precipitate a more fundamental change in the vector population dynamics [35] either through an outdoor behavioural shift in the local vector population or via vector population replacement. Indeed *An. gambiae* mosquitoes have been reported seeking hosts in outdoor venues as much as indoors in the Punta Europa region of Bioko Island [36]. Likewise, the widespread use of organophosphates and synthetic pyrethroids for the control of *Ae. aegypti* and *Culex spp*. has fed the emergence of insecticide resistance in many dengue [37] and filariasis [38] endemic countries. Although it is too early to draw strong conclusions on the behavioural effects of such a sustained insecticide use, it is clear that new tools are needed to tackle outdoor transmission. Thus, new initiatives such as AVECNET (www.avecnet.eu) are vital to help develop and evaluate new vector control systems for malaria and other mosquito-transmitted diseases.

**Table 1.** WHO-approved insecticides for adult malaria mosquito control. Their applications and P450 resistance mechanisms [26].

dates	insecticide	type^a^	application		P450 resistance^b^
			IRS	ITN	
1940–1945	DDT	OC	X		r
1946–1950	Lindane	OC			
1951–1955	Malathion	OP	X		r
1961–1965	Fenitrothion	OP	X		r
	Propoxur	Ca	X		r
1966–1970	Chlopyriphos-methyl	OP	X		r
1971–1975	Pirimiphos-methyl	OP	X		r
	Bendiocarb	Ca	X		r
	Permethrin	Pyr	X	X	R
1976–1980	Cypermethrin	Pyr	X	X	R
1981–1985	Alpha-cypermethrin	Pyr	X	X	R
	Cyfluthrin	Pyr	X	X	R
	Lambda-cyhalothrin	Pyr	X	X	R
	Deltamethrin	Pyr	X	X	R
	Bifenthrin	Pyr	X	X	R
1986–1990	Etofenprox	Pyr	X	X	R

^a^OC, organochlorine; OP, organophosphate; Pyr, pyrethroid; Ca, carbamate.

^b^*r*, weak contribution to resistance; R, major contribution to resistance.

## 4. P450s and insecticide resistance

The mechanisms responsible for insecticide resistance are complex and include behavioural and/or physiological changes of mosquitoes leading to insecticide avoidance, altered penetration, sequestration, target site alteration or bio-degradation. In mosquitoes, resistance is mainly associated with target site modification and metabolic resistance. Target site resistance involves mutations leading to well-defined target site alteration and resistance to chemical insecticides [39]. Metabolic resistance, on the other hand, involves more subtle alterations in the expression of a complex array of enzymes and detoxification pathways [7,40], the mechanisms of which are far less well understood.

Metabolic resistance occurs through increased bio-degradation of the insecticide, usually through overproduction of detoxification enzymes such as P450s, glutathione S-transferases (GSTs) and carboxy/cholinesterases (CCE) [40]. Of these, P450s are the primary enzyme family associated with resistance to most insecticides including pyrethroids, the most widely used class of insecticide for vector control. Elevated levels of P450 activity are frequently observed in pyrethroid-resistant malaria vectors in Africa [29,41–48]. Esterase hydrolysis of pyrethroids leading to detoxification is also believed to act as a cause of metabolic resistance in some instances [42,49], while GSTs are regularly found overexpressed in pyrethroid-resistant strains [50]. However, the contribution these enzymes make towards pyrethroid resistance and their biochemical relationships with P450-mediated resistance is still unclear.

## 5. Identification of P450s involved in resistance

Early detection of resistance is critical for sustainable management of the few insecticides available, for example, via removal of an insecticide before resistance becomes fixed in the population to allow recovery of the susceptible phenotype [33,51]. Following the development of a highly selective *An. gambiae* detox-chip [52], microarray-based studies have helped us identify several P450s in *An. gambiae* associated with pyrethroid resistance (table 2). These include CYP6Z1, CYP6Z2, CYP6M2, CYP6P3 and CYP325A3 [48,52,60,61]. Of these, CYP6P3 and CYP6M2 have appeared most widely over-transcribed in resistant field populations [47,48]. Importantly, they have been shown to metabolize permethrin and deltamethrin [53,54], thus operationally are considered key diagnostic markers of resistance. Most recently, high levels of *CYP6M2* gene expression have been found in a highly DDT-resistant population of *An. gambiae* from Ghana, using a novel whole genome microarray [55]. Likewise, CYP6Z1 has been found overexpressed in both pyrethroid and DDT-resistant strains, and shown to metabolize DDT [56], placing it in the frontline of metabolic resistance markers for DDT. Finally, CYP6Z2 [62] is an interesting P450 as it has been reported overexpressed in Ghanaian strains of permethrin-resistant *An. gambiae* [60]. However, it does not appear to metabolize pyrethroids [62]. Whether it is an artefact or has a physiological involvement in insecticide clearance is not yet known. Our view is the latter since recent experiments (Achandor-Proust, M. J. I. Paine and J.-P. David 2012, unpublished work) have shown that CYP6Z2 and its *Ae. aegypti* orthologue CYP6Z8 are capable of metabolizing 3-phenoxybenzoic alcohol and 3-phenoxybenzaldehyde, common pyrethroid metabolites produced by carboxylesterases.

**Table 2.** African anopheline, P450s associated with insecticide resistance. P450s in bold have been heterologously expressed and validated as insecticide metabolizers.

P450	species	location	insecticide	reference
**CYP6P3**	*An. gambiae*	Ghana (Dodowa); Benin (Akron and Gbedjromede); Nigeria (Orogun)	Permethrin and Deltamethrin	[47,48,53]
**CYP6M2**	*An. gambiae*	Ghana (Odumasy and Dodowa; Great Accra); Benin (Akron and Gbedjromede); Nigeria (Orogun)	Deltamethrin DDT	[47,54,55]
**CYP6Z1**	*An. gambiae*	Kenya (Kisumu ZANU)	DDT	[52,56]
CYP314A1	*An. gambiae*	Zanzibar, Tanzania (ZAN/U strain)	DDT	[57]
CYP12F1	*An. gambiae*	Zanzibar, Tanzania (ZAN/U strain)	DDT	[52]
CYP325A3	*An. gambiae*	Western Kenya (Kisumu region)	Permethrin	
CYP6Z3	*An. gambiae*	Ghana (Odumasy)	Permethrin	[48]
CYP325A3	*An. gambiae*	Nigeria (Ipokia)	Permethrin	[58]
CYP4G16	*An. arabiensis*	Northern Cameroon	Deltamethrin	[53]
CYP4H24	*An. arabiensis*	Northern Cameroon	Deltamethrin	[53]
CYP325C2	*An. arabiensis*	Northern Cameroon	Deltamethrin	[53]
CYP6AG1	*An. arabiensis*	Northern Cameroon	Deltamethrin	[53]
**CYP6P9**	*An. funestus*	Mozambique	Permethrin, Deltamethrin	[45,59]

As malaria can be transmitted by more than 30 species of *Anopheles* [63], there is considerable interest in identifying resistance mechanisms in other species as well. In *An. funestus,* the second major vector of malaria in Sub-Saharan Africa, the use of quantitative trait loci (QTL) has identified CYP6P9, CYP6P4, CYP6Z1, CYP6Z3 and CYP6M7 as being strongly associated with pyrethroid resistance [44,64] (table 2). CYP6Z1 and CYP6Z3 have already been linked with insecticide resistance in *An. gambiae*, while CYP6P9 and CYP6M7 are orthologues of *An. gambiae* resistance markers, CYP6P3 and CYP6M2, respectively.

To date, increased levels of CYP6P9 have been most frequently observed in pyrethroid-resistant laboratory and field populations from Mozambique [44–46,65,66], Uganda [67] and Benin [68]. Furthermore, it has been suggested that these elevated P450s also confer cross-resistance to carbamates [41]. Interestingly, *CYP6P9* and *CYP6P4*, two major candidate genes associated with resistance to pyrethroids, are both duplicated [44], with relatively extensive sequence variation (94% nucleotide sequence similarity for *CYP6P4a* and *b* and 95% similarity for *CYP6P9a* and *b*). Recent transgenic overexpression of CYP6P9a and CYP6P9b in *Drosophila melanogaster* has demonstrated that each copy of the duplicated CYP6P9 can confer resistance to pyrethroid [59]. Therefore, the simultaneous overexpression of CYP6P9a and CYP6P9b in field populations is likely to increase their resistance level. We have also expressed *CYP6P9a* and *CYP6P9b* in *E. coli* and found them to be capable of metabolizing permethrin and deltamethrin [59], confirming their status as key metabolic resistance genes in *An. funestus*.

In the Asian malaria vector *An. minimus* two P450s, CYP6P7 and CYP6AA3, have been shown to metabolize the pyrethroids permethrin, cypermethrin and deltamethrin but not bioallethrin [69–71]. Furthermore, in contrast to CYP6AA3, CYP6P7 had limited capacity for λ-cyhalothrin (a pyrethroid used extensively for IRS). From an operational perspective, such differences in pyrethroid specificity show that cross-resistance cannot be easily extrapolated even within a class of insecticides. Importantly, although these P450s are found overexpressed in laboratory-resistant strains exposed to pyrethroids [69,70,72], a firm association with pyrethroid resistance in field populations has yet to be established.

In *Ae. aegypti*, a number of studies have been conducted to investigate insecticide resistance in different field or laboratory populations [73–81]. As with *An. gambiae*, numerous different P450s were found over-transcribed in resistant populations or laboratory strains, often belonging to tight genomic clusters resulting from recent duplications (table 3). For example, the CYP9J subfamily showed the strongest increased diversity compared with *An. gambiae* and other insects possibly reflecting an adaptation to environmental xenobiotics [82]. Of these, CYP9J32 stands out as being over-transcribed in deltamethrin- and permethrin-resistant strains in Thailand, Mexico and Vietnam [78,82]. Interestingly, while CYP9J32 is capable of metabolizing both these pyrethroids [83], it shows a distinct preference for deltamethrin. CYP9J24, CYP9J26 and CYP9J28 are other *Ae. aegypti* P450s that have been shown to metabolize pyrethroids [83], thus elevated levels of one or more of these P450s in field populations of mosquitoes should be considered indicators of metabolic resistance to pyrethroids.

**Table 3.** *Aedes* P450s associated with insecticide resistance. P450s in bold have been heterologously expressed and validated as pyrethroid metabolizers.

P450	species	location	insecticide	reference
**CYP9J32**	*Ae. aegypti*	Mexico (Isla Mujeres); Thailand (Ban Pang Mai Dang); Vietnam (Nha Trang city, Khanh Hoa province, Southern)	Permethrin, Deltamethrin	[78,82]
**CYP9J24**	*Ae. aegypti*	Mexico (Isla Mujeres)	Permethrin	[82]
**CYP9J28**	*Ae. aegypti*	Mexico (Isla Mujeres)	Permethrin	[82]
CYP12F6	*Ae. aegypti*	Mexico (Isla Mujeres)	Permethrin	[82]
CYP304C1	*Ae. aegypti*	Thailand (Ban Pang Mai Dang)	Permethrin	[82]
CYP6CB1	*Ae. aegypti*	Thailand (Ban Pang Mai Dang)	Permethrin	[82]
CYP6M6	*Ae. aegypti*	Martinique Island	Deltamethrin	[80,81]
CYP6M10	*Ae. aegypti*	laboratory strain	Permethrin	[79]
CYP6M11	*Ae. aegypti*	laboratory strain	Permethrin	[79]
CYP6Z6	*Ae. aegypti*	Martinique Island	Deltamethrin	[80,81]
CYP6Z8	*Ae. aegypti*	Martinique Island	Deltamethrin	[80,81]
CYP6F3	*Ae. aegypti*	laboratory strain	Permethrin	[79]
CYP9J10	*Ae. aegypti*	Thailand (Ban Pang Mai Dang)	Permethrin	[82]
CYP9J10	*Ae. aegypti*	Mexico (Isla Mujeres)	Permethrin	[82]
CYP9J19	*Ae. aegypti*	Mexico (Isla Mujeres)	Permethrin	[82]
CYP9J22	*Ae. aegypti*	Martinique Island	Deltamethrin	[80,81]
CYP9J23	*Ae. aegypti*	Martinique Island	Deltamethrin	[81]
CYP9J26	*Ae. aegypti*	Thailand (Ban Pang Mai Dang)	Permethrin	[82]
CYP9J27	*Ae. aegypti*	Mexico (Isla Mujeres); Thailand (Ban Pang Mai Dang)	Permethrin	[82]

P450s have long been recognized as playing a key role in pyrethroid resistance in *Culex* spp. [84,85]. In *C. quinquifasciens*, four P450s have emerged as candidates for permethrin resistance: *CYP6AA7*, *CYP9J40*, *CYP9J34* and *CYP9M10,* with overexpression levels closely correlated to their levels of resistance in laboratory strains from Alabama, USA (table 4). The overexpression of CYP9M10 has also been reported in a laboratory-resistant *Culex* mosquito strain established from Saudi Arabia [137], that also metabolizes permethrin and deltamethrin [86], strengthening links with pyrethroid resistance in *Culex* mosquitoes [87–89]. Interestingly, a partial *CuRE1* (*Culex* Repetitive Element 1) transposable element has been implicated as a *cis*-regulatory element driving up expression of the CYP6M10 [86]. Furthermore, the presence of the *CuRE1* insertion demonstrates a strong association with permethrin resistance (odds ratio = 22.4 for *CuRE1* homozygotes) in field populations from Accra, Ghana, as well as in Malawi, Zambia, Mozambique and Uganda, indicating that this resistance mechanism is widespread throughout Africa.

**Table 4.** *Culex* P450s associated with insecticide resistance. P450s in bold have been heterologously expressed and validated as pyrethroid metabolizers.

P450	species	location	insecticide	reference
**CYP6M10**	*C. quinquifasciatus*	USA (Huntsville HamCq strain); Saudi Arabia (ISOP450 strain); Japan (ISOJPAL strain)	Permethrin, Deltamethrin	[26,57,58,86–133]
**CYP4H34**	*C. quinquifasciatus*	Saudi Arabia (ISOP450 strain); Japan (ISOJPAL strain)	Permethrin	[86,87,89]
CYP6AA7	*C. quinquifasciatus*	USA (Huntsville and Mobile, HAmCq and MAmCq strains respectively)	Permethrin	[133,134]
CYP4C5W1	*C. quinquifasciatus*	USA (Huntsville and Mobile, HAmCq and MAmCq strains respectively)	Permethrin	[133,134]
CYP9J40	*C. quinquifasciatus*	USA (Huntsville HamCq strain)	Permethrin	[133]
CYP9J34	*C. quinquifasciatus*	USA (Huntsville HamCq strain)	Permethrin	[133]
CYP6F1	*C. pipiens*	China (Shanghai Insect Institute strain)	Deltamethrin	[135]
CYP4H21	*C. pipiens*	China (Shanghai Insect Institute strain)	Deltamethrin	[136]
CYP4H22v1	*C. pipiens*	China (Shanghai Insect Institute strain)	Deltamethrin	[136]
CYP4J4v2	*C. pipiens*	China (Shanghai Insect Institute strain)	Deltamethrin	[136]
CYP4J6v1	*C. pipiens*	China (Shanghai Insect Institute strain)	Deltamethrin	[136]
CYP4J6v2	*C. pipiens*	China (Shanghai Insect Institute strain)	Deltamethrin	[136]

## 6. Characterization of P450-mediated insecticide degradation mechanisms

While the biochemical and pharmacological pathways of human P450 drug metabolism are well mapped, very little is known about the equivalent insect mechanisms of insecticide breakdown. For instance, the metabolic pathways of pyrethroid degradation in whole, or partially extracted, insects have extensively been examined (for review see [90]), however, metabolism by individual insect P450s has not been particularly well studied. Such information is fundamental to our understanding of the metabolic fate of insecticides and the design of new molecules to overcome or inhibit resistance. Recent work has shown 3-phenoxybenzaldehyde to be a major product of CYP6AA3-mediated deltamethrin metabolism [69], while more detailed analysis of deltamethrin metabolism by *An. gambiae* CYP6M2 [53] has revealed several metabolite products associated with multiple binding modes. The major route of metabolism was 4′-hydroxylation, which is common in insect P450s [90] presumably rendering the molecule more excretable and less toxic. However, sequential breakdown of 4′-hydroxydeltamethrin into several minor products also occurs. Whether these play a role in insecticide resistance is a moot point. The secondary and tertiary metabolism of deltamethrin by CYP6M2 is likely to influence the reaction kinetics through competitive effects and may even inactivate the enzyme through production of reactive metabolites such as quinone or cyanide. Finally, it is probable that some mosquito P450s are also involved in the further processing of common pyrethroid metabolites such as 3-phenoxybenzaldehyde and 3-phenoxybenzyl alcohol, and could therefore contribute to resistance. Further research is clearly required to understand more precisely the pharmacology of insecticide metabolism in mosquitoes, particularly in the light of P450s such as CYP6Z2 that are linked with pyrethroid resistance, yet do not metabolize these insecticides.

## 7. Cross-response mechanisms between insecticides and pollutants

Most molecular studies related to P450-mediated insecticide resistance have focused on the strong selection pressure caused by insecticides, while less attention has been paid to the impact of the environment on the modulation of P450-mediated response to insecticides. Although exposure to agricultural insecticides is widely linked to the selection of resistance in disease vectors [47,48,91,92], the possible role of pollutants in the selection and/or modulation of P450-mediated resistance mechanisms is less clear. This has important implications for resistance management and the efficacy of vector control activities. For example, if the selection pressure is coming from alternative sources, then rotational use of different classes of insecticides will be far less effective in IRS programmes. Similarly, if particular pollutants are strong inducers of P450s involved in resistance, the use of insecticides in areas highly polluted with those chemicals may be less efficient, thus requiring higher doses of insecticides. Indeed, with the increasing reliance on pyrethroids for malaria control, the ‘hidden impact’ of anthropogenic pollutants on mosquito ecosystems is an important question to address.

Since pyrethroids and many other synthetic insecticides closely resemble plant allelochemicals, it has been suggested that P450s responsible for insecticide detoxification might have evolved from those responsible for allelochemical detoxification [93–96]. Considering the capacity of P450s to respond to a wide range of xenobiotics through induction/repression mechanisms [7], interactions between natural or anthropogenic xenobiotics found in mosquito habitats and P450-mediated tolerance/resistance might be expected. Thus, residual herbicides and insecticides from agriculture as well as pollutants such as polycyclic aromatic hydrocarbons (PAHs), polychlorinated biphenyls (PCBs), dioxins, heavy metals or drugs are likely to interact with P450s. Certainly, laboratory studies have pointed out an increased tolerance of mosquitoes to insecticides following pollutant exposure [138]. Pre-exposure of *Ae. albopictus* to the major leachate compound benzothiazole (BZT) and its derivatives from automobile tyres (an important breeding habitat), produced mosquito larvae that were more tolerant to carbaryl, rotenone and temephos [139]. More recently, the use of DNA microarrays has shown that *Ae. aegypti* larvae exposed to the herbicides atrazine and glyphosate, the PAHs fluoranthene and benzo[a]pyrene, and the metal copper displayed an increased tolerance to insecticides, which was linked to the induction of various P450s including particular ones frequently associated with pyrethroid resistance [97–100]. Deep sequencing of cDNA libraries from larvae exposed to pollutants (copper, flouranthene and atrazine) and insecticides (propoxur, permethrin and imidacloprid) has confirmed the cross-induction of detoxification genes by insecticides and pollutants, with particular P450s induced by multiple xenobiotics [97]. More recent laboratory experiments combining exposure to a sub-lethal dose of the PAH fluoranthene and subsequent selection with the pyrethroid permethrin for several generations indicates that pollutants may modulate the selection of particular detoxification enzymes by insecticides [79]. Overall, evidence is accruing that pollutants can increase the tolerance of mosquitoes to insecticides or modulate the selection of inherited detoxification mechanisms, although the impact of complex pollutant mixtures requires further research. Although these studies evidenced the links existing between anthropogenic xenobiotics and insecticide resistance, the complex role of mosquito P450s and their relationships with environmental xenobiotics and insecticides have yet to be resolved.

## 8. P450s and sustainable use of insecticides

The use of dichlorodiphenyltrichloroethane (DDT) exemplifies the problems of sustainable management of insecticides. The discovery of the insecticidal property of DDT by Paul Hermann Müller in 1939 marked a watershed in vector control [101], leading to malaria elimination in several countries including the USA [102–106]. However, the indiscriminate use of DDT in agriculture and its environmental persistence (half-life of up to 10 years) led to its worldwide ban for all general use apart from vector control [107,108]. Ultimately, intensive efforts between 1955 and 1969 to eradicate malaria foundered on the development of resistance in anopheline mosquitoes [108].

Insecticides need to be tightly managed to ensure adequate dosing without causing danger to health and environment or inducing resistance. Current eradication efforts, which are focused on the use of insecticides in materials (bednets and wall-hangings) or targeted house spraying, minimize to some extent the environmental impact of indiscriminate application. Nevertheless, resistance remains problematic and sustainable vector control is limited with only four available classes of insecticide. This has encouraged the reintroduction of DDT as a front-line insecticide [109], while the World Health Organization has begun a global strategy to tackle the resistance problem [110], advocating the need for more systematic and sophisticated surveillance systems for resistance, plus new classes of insecticides.

At present, biochemical assays for detecting metabolic resistance by P450s are in general use [8], but they usually employ generic haem peroxidase assays that are recognized by many members of the enzyme family [111], compromising sensitivity and specificity. However, with the identification of P450s associated with insecticide resistance, the development of more specific diagnostic assays is now feasible. For example, luciferin substrates have been used for tracking general P450 activity in the mosquito *C. pipiens* [112], while most recently the screening of individual P450s against fluorogenic and luminescent compounds has identified a further set of potential diagnostic substrates for pyrethroid-metabolising P450s in *Ae. aegypti* and *An. gambiae* [83]. Luciferin-PPXE in particular is preferentially metabolized by at least three major pyrethroid metabolizers, *Ae. aegypti* CYP9J32, and *An gambi*ae CYP6M2 and CYP6P3, thus a candidate probe for monitoring P450-mediated resistance phenotypes. Ultimately, the accumulation of resistance markers will strengthen operational surveillance, thus enabling reactive measures to be taken at an early stage.

Under the umbrella of the Innovative Vector Control Consortium (IVCC), a public–private venture set up in 2005, a range of new initiatives are now underway to develop new insecticides and monitoring tools [27]. For example, high- throughput DNA-based diagnostics are being developed for a Vector Population Monitoring Tool (VPMT) that can be used on dead mosquito collections, passively collected in fixed window traps by householders, to monitor mosquito population densities, their species, insecticide resistance status and malaria sporozoite infection rates [113]. Development of these simple diagnostics has been driven by the availability of the *An. gambiae* genome sequence. To date, the programme has enlisted state-of-the-art molecular techniques, genomics, single nucleotide polymorphism (SNP) mapping, QTL and association mapping coupled to metabolism studies to identify the genes responsible for the insecticide resistance phenotype in the mosquitoes. Linking these resistance genes to SNP markers is the ultimate goal as this allows them to be tracked using simple PCR reactions in the field. Notwithstanding the inherently high frequency of SNPs in mosquitoes discussed further on, SNP markers for infection status, species identification and target–site mutations conferring pyrethroid resistance are now available for *An. gambiae* [113–116], and a programme to develop similar markers is underway for *An. funestus* [65,117].

## 9. P450s and insecticide design

Greater efforts are now being directed towards the development of new insecticides, and with it a growing need to take into account P450 activity in the design process. For example, in the same way that liver P450s influence drug metabolism in humans, insect P450s can influence the metabolism and disposition of insecticides. Thus, understanding which insecticides are metabolized by what P450s is important to delineate potential strengths and liabilities of insecticide compounds. Until now this has been impossible owing to a lack of knowledge about insect-metabolizing P450s. However, as more mosquito P450s associated with insecticide metabolism are identified, these can be used to develop *in vitro* assays for the design of insecticides including biochemical characterization of insecticide metabolism and high-throughput screening of P450 liabilities to guide the development of vector control compounds.

In humans, it is well known that only a handful of their 57 P450s (CYPs 3A4, 2D6, 2C9, 1A2 and 2C19) are responsible for the metabolism of most drugs in clinical use [118]. Consequently, interactions with these enzymes underpin drug development. What are the major insect P450s involved in insecticide metabolism, and can they be similarly used for development of new vector control tools? In *Drosophila melanogaster* the overexpression of a single P450 allele (CYP6G1) can confer resistance to DDT [119]. Similarly, CYP6D1 plays a predominant role in permethrin resistance in house-flies [120], while CYP6BQ9 seems responsible for the majority of deltamethrin resistance in *Tribolium castaneum* [121]. In mosquitoes, however, numerous P450s are associated with insecticide resistance (tables 2–4), although relatively few have been functionally validated.

Identifying a core set of insecticide-metabolizing P450s equivalent to human drug-metabolising P450s is further complicated by the extremely high frequency of SNPs in mosquitoes. Major resequencing of pooled genomes from geographically diverse *An. gambiae* has revealed, on average, a SNP every 34 bp, approximately 10-fold higher than humans [122]. Furthermore, SNPs were significantly more frequent in members of the P450 and carboxy/cholinesterase gene families [122], possibly reflecting less stringent selection owing to flexibility in function among detoxifying enzymes. Indeed, a similar differentiation between P450s with xenobiotic and endogenous substrate preferences has been observed with gene duplication events. For example, stable genes are characterized by few or no gene duplications or losses in species, whereas unstable genes are characterized by frequent gene duplications and losses even among closely related species [123]. Thus, considering the large expansion of the P450 gene family in mosquitoes*, Ae. aegypti* in particular [82], gene conversion offers another mechanism for rapid evolution of new sequences [7,124]. For example, CYP6Z2 and CYP6Z3, located adjacent on the chromosomal arm 3R in *An. gambiae*, are 93.5 per cent identical and presumably the result of a recent duplication event [61]. However, the sequences show a particularly high degree of homology (approx. 100%) within the first 600 bases, which may signify a gene conversion event.

Further biochemical and structural information on the impact of allelic variants on P450 function is needed to increase our molecular understanding of insecticide metabolism. As yet, crystal structures of insect P450s have not been resolved, although *in silico* homology modelling provides useful insight into the molecular basis of insecticide binding [54,56,62,125–128]. For example, molecular modelling, supported by enzyme characterization, has been particularly effective in deciphering the catalytic site geometry of CYP6Z1 and the mechanism of DDT metabolism [56]. Bearing in mind the high degree of allelic variance in mosquito P450s [122], such models are particularly suitable for the systematic analysis of allelic variants to understand resistance mechanisms and aid design of new insecticides. Finally, such an approach may also be of interest for identifying new P450 candidates from other mosquito species showing active sites highly similar to known insecticide metabolizers.

Recent advances in whole genome sequencing, transcriptomic and proteomic analysis has allowed fine-scale genetic comparisons across species and populations as recently reviewed [129]. This is starting to filter into field-based studies such as the Malaria Genomic Epidemiology Network (MalariaGEN), a community of researchers in more than 20 countries who are working together to understand how genome variation in human, *Plasmodium* and *Anopheles* populations affect the biology and epidemiology of malaria (http://www.malariagen.net/). In addition, data from many other transcriptomic studies are highlighting a key role for P450 in insecticide detoxification in mosquitoes [130] and other insect pests such as the whitefly [131] and cockroach [132].

## 10. Conclusion

Although the involvement of P450s in insecticide resistance has been known since the 1980s, identifying mosquito P450s involved in the metabolism of insecticides has been a challenging task in the last decades because of insufficient genomic resources and technical limitations. This has probably led to the underestimation of the impact of metabolic resistance mechanisms on vector control strategies. Since the sequencing of mosquito genomes, tremendous progress has been made in this research field with several candidate CYP genes now identified and validated as insecticide metabolizers. Although a lot remains to be done, as more and more mosquito P450s are characterized, we start to get a better view of the role of mosquito CYPomes in their adaptation to environment and insecticides. Supported with recent technical advances, this research area opens up new perspectives for the control of vector-transmitted tropical diseases and the sustainable use of insecticides on Earth.
